# Development of an evidence-based diagnostic algorithm for infection in patients with transcutaneous osseointegration following amputation

**DOI:** 10.5194/jbji-9-49-2024

**Published:** 2024-02-06

**Authors:** Shafaf Hasin Alam, Jason S. Hoellwarth, Kevin Tetsworth, Atiya Oomatia, Tristen N. Taylor, Munjed Al Muderis

**Affiliations:** 1 Princess Alexandra Hospital, 199 Ipswich Rd, Woolloongabba, Queensland, 4102, Australia; 2 Limb Lengthening and Complex Reconstruction Service, Osseointegration Limb Replacement Center, Hospital for Special Surgery, 535 East 70th Street, New York, NY, 10021, USA; 3 Department of Orthopaedic Surgery, Royal Brisbane and Women's Hospital, Queensland, Australia; 4 Limb Reconstruction Centre, Macquarie University Hospital, Macquarie University, Macquarie Park, Australia; 5 Baylor College of Medicine, Houston TX. 1 Baylor Plaza, Houston, TX 77030, USA

## Abstract

**Introduction**: Transcutaneous osseointegration following amputation (TOFA) confers better mobility and quality of life for most patients versus socket prosthesis rehabilitation. Peri-TOFA infection remains the most frequent complication and lacks an evidence-based diagnostic algorithm. This study's objective was to investigate preoperative factors associated with positive intraoperative cultures among patients suspected of having peri-TOFA infection in order to create an evidence-based diagnostic algorithm. **Methods**: We conducted a retrospective study of 83 surgeries (70 patients) performed to manage suspected lower-extremity peri-TOFA infection at a specialty orthopedic practice and tertiary referral hospital in a major urban center. The diagnosis of infection was defined as positive intraoperative cultures. Preoperative patient history (fevers, subjective pain, increased drainage), physician examination findings (local cellulitis, purulent discharge, implant looseness), and laboratory data (white blood cell count, C-reactive protein (CRP), erythrocyte sedimentation rate (ESR), and external swab culture) were evaluated for association with subsequent positive intraoperative cultures using regression and area under receiver–operator curve (AUC) modeling. **Results**: Peri-implant limb pain (highly correlated with infection), ESR >30 (highly correlated against infection), positive preoperative swab (moderately correlated with infection), gross implant motion (moderately correlated against infection), and erythema or cellulitis of the transcutaneous region (mildly correlated with infection) were variables included in the best AUC model, which achieved an 85 % positive predictive value. Other clinical findings and laboratory values (notably CRP and WBC) were non-predictive of infection. **Conclusions**: This seminal investigation to develop a preoperative diagnostic algorithm for peri-TOFA infection suggests that the clinical examination remains paramount. Further evaluation of a wider spectrum of clinical, laboratory, and imaging data, consistently and routinely collected with prospective data techniques in larger cohorts of patients, is necessary to create a robust predictive algorithm.

## 1 Introduction

Lower-limb amputation poses significant mobility and quality of life challenges to patients (Butler et al., 2014; Samuelsson et al., 2012). The socket prosthesis (SP) is the current standard rehabilitation option for patients following amputation (Esquenazi, 2004). However, socket–skin issues are associated with high rates of dissatisfaction and abandonment due to issues including discomfort, chronic skin irritation, excessive sweating, and prosthesis fit (Murray and Forshaw, 2013; Baars et al., 2018).

**Figure 1:**
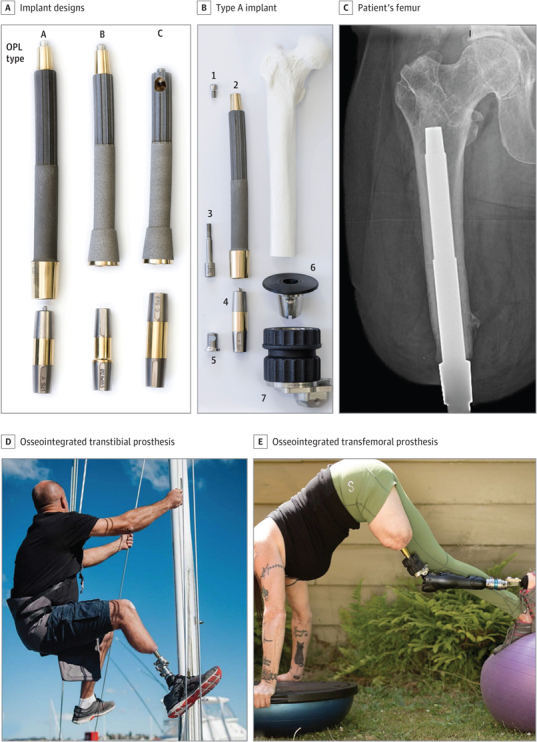
Osseointegration implant and patient examples. **(a)** Photograph of common osseointegrated prosthetic limb (OPL – Permedica) implant designs. The top portion is made of titanium with a textured intramedullary portion and a smooth transcutaneous portion and is implanted into the patient's residual limb. The dual cone below the implant is a connector to the external prosthetic limb. **(b)** Exploded view of a type-A implant with the components arranged at approximately the proximal–distal levels in which they would be assembled and implanted in a patient who had undergone a femoral amputation. The numbers in **(b)** indicate the following: 1 – proximal cap screw, 2 – OPL body, 3 – safety screw, 4 – dual-cone transcutaneous adapter, 5 – prosthesis adapter screw, 6 – proximal connector, and 7 – prosthetic connector. **(c)** Anterior–posterior radiograph of a TOFA implant in a patient's femur. The textured portion is intramedullary; the smooth portion is transcutaneous. Panels **(d)** and **(e)** are examples of activities that a patient with an osseointegrated transtibial (**(d)** or transfemoral **(e)**) implant can achieve with a skeletally connected prosthesis. Figure reused with permission from Hoellwarth et al. (2022a).

Transcutaneous osseointegration following amputation (TOFA), previously reviewed (Hoellwarth et al., 2020a, 2022c), is a rehabilitation alternative (Fig. 1). By eliminating the socket, the direct skeletal connection to the prosthetic limb offers superior mobility and quality of life (Kunutsor et al., 2018; Hebert et al., 2017). Complications following TOFA include periprosthetic fracture (approximately 6 % of osseointegrated transfemoral patients) (Hoellwarth and Rozbruch, 2022; Hoellwarth et al., 2020b; Örgel et al., 2021), while the associated mortality is below 1 % (Hoellwarth et al., 2022a). The most common important complication is infection prompting surgical intervention, occurring in 5 %–8 % of patients (Tropf and Potter, 2023).

There is no consensus regarding the diagnosis and treatment of peri-TOFA infection despite multiple classifications of the extent of intervention rendered (Al Muderis et al., 2016; Hoellwarth et al., 2022d). Clinicians assessing osseointegrated patients with a concern of infection rely on personal judgment, generally influenced by prosthetic joint infection (PJI) principles, despite the distinction that osseointegration features permanently exposed implants through a transcutaneous skin portal. An evidence-based, highly sensitive and specific diagnostic criteria for peri-TOFA infection, such as has been developed for PJI (Shohat et al., 2019), is necessary to render more uniform and improved care.

This study aims to address that knowledge gap. The primary aim is to propose a diagnostic algorithm of peri-TOFA infection based on preoperative patient history, clinical examination, serological data, and peri-portal bacterial culture swabs.

## 2 Methods

### 2.1 Ethics approval

This study was approved by the Institutional Research Ethics Committee.

### 2.2 Patient selection

Retrospective review of our group's prospectively maintained osseointegration registry was performed to identify patients who had additional surgery post-TOFA to manage suspected infection of either or both soft tissue and/or bone. The general indication criteria for patients considering TOFA have been previously described (Al Muderis et al., 2017; Haque et al., 2020), and techniques have been demonstrated (Hoellwarth et al., 2022b; Geiger et al., 2022). Generally, patients considered for osseointegration are skeletally mature adults who either (1) report pain or mobility dissatisfaction with their SP; (2) have an intact limb with incapacitating pain, complex deformity, or profound distal weakness and whose functional capacity is considered likely to be improved by amputation; or (3) are recent amputees preferring osseointegration to SP rehabilitation. Contraindications to osseointegration are modifiable comorbidities which may impair successful bone and/or wound healing, such as active infection or malignancy, though upon treatment or optimization of those modifiable comorbidities, these patients may be considered to be suitable.

Between 2010–2021, 792 patients had transfemoral and/or transtibial osseointegration at our primary practice location. The inclusion criterion for this study was any patient who had subsequent surgery to address suspected infection. Patients without additional post-TOFA surgery were excluded. Patients who had surgery for periprosthetic fractures were also excluded. All implant revisions were included in this study as a loose implant could allow bacterial ingress through the portal.

This identified 70 patients who had 83 total subsequent surgeries (52 debridement and implant retention (DAIR), 31 removals), all with intraoperative culture swab positivity data available. The indications for surgical intervention were made based on the surgeon's judgment of patient-provided history, clinical examination, laboratory data, and/or peri-portal swab. Notably, imaging was excluded from this study's evaluation (plain radiographs, computed tomography, and magnetic resonance imaging) because different osseointegration implants were used, some of which have designs which do not achieve distal bone–implant integration, necessarily resulting in confounding radiographic and advanced imaging patterns.

### 2.3 Infection surgery techniques

The decision for DAIR versus implant removal surgery was not algorithmic but instead based on surgeon judgment. Factors which generally led to DAIR included an undamaged implant that was stable prior to and during surgery, with a healthy-appearing bone–implant interface; any implant-related pain was short term; and the patient had not had multiple prior debridements. Factors more strongly prompting implant removal included broken implants, implants that were loose before or during surgery, unhealthy appearance of the bone–implant interface, implant-related pain that was long term, or multiple prior debridements. The duration of pain or the number of DAIRs before removal were subject to surgeon judgment. At least four culture samples were taken at any surgery with a concern of infection, either from extramedullary locations (for DAIR) or extra- and intramedullary locations (for removals).

### 2.4 Developing the algorithm

The 83 surgical episodes were dichotomized into infected and aseptic cohorts based on whether at least one surgically obtained culture from the set of samples grew bacteria. The definition was set very conservatively (excluding potential additional definitions such as malodorous or purulent drainage or sinus tract) for two specific reasons. First, to minimize type-1 error (an incorrect presumption of infection). Second, to minimize the possible clinical bias of performing surgery for superficial cellulitis (which might be sufficiently treated by antibiotics without surgery) that may sometimes induce edematous drainage through the transcutaneous site, leading to misdiagnosis of deep infection.

**Table 1:** Patient demographics.

	Infected (57)	Aseptic (26)	p=
Age (years)	54.3±13.3	50.8±10.6	.857
Male	36 (63 %)	14 (54 %)	.473
BMI (kg m^-1^)^*^	26.0±4.4	25.1±4.9	.425
Diabetes	6 (11 %)	1 (4 %)	.425
Smoking	1 (2 %)	1 (4 %)	.531
Femur	28 (49 %)	17 (65 %)	.235
Tibia	29 (51 %)	9 (35 %)	.235

^*^ BMI refers to body mass index, adjusted for level of amputation based on amputee coalition https://www.amputee-coalition.org/limb-loss-resource-center/resources-filtered/resources-bytopic/healthy-living/about-bmi/ (last access: 31 January 2024).

### 2.5 Statistical analysis

The following analyses were performed to identify which suspected signs, symptoms, and laboratory markers had relevance to infection prediction. The dependent variable (outcome) of interest for all analyses was a positive culture taken during surgery. First, simple logistic regression modeling was performed for each independent variable to determine associations of single risk factors with positive intraoperative cultures. Then, multivariable logistic regression was performed to determine simultaneous risk factor associations in four scenarios: first, considering only the variables identified as significant in the univariate analysis and also other sensible clinical variables (Bursac et al., 2008; Heinze et al., 2018); second, considering all the clinical examination data, without laboratory or swab data, to simulate a pure clinical judgment; third, considering only the three laboratory markers (erythrocyte sedimentation rate (ESR), C-reactive protein (CRP), and white blood cells (WBCs)) to simulate a decision made based on a patient who may be unavailable for skilled physician evaluation but has access to a local medical laboratory; and fourth, considering the laboratory markers and also a swab of the transcutaneous site to simulate a patient who has access to a primary physician who can order labs and procure a swab but may not be able to provide skilled clinical assessment.

An optimal multivariable model was obtained by stepwise approach, yielding the smallest Akaike's Information Criterion (AIC; an indicator of the model's predictive ability). Sensible clinical variables were considered, even if not they were individually modeled as statistically significant, to produce the most appropriate model (Bursac et al., 2008; Heinze et al., 2018). Risk factor logistic regression coefficients were proportionally scaled to yield score coefficients.

To determine the most reliable model, patient data were input to each model, and the area under the curve (AUC) of a receiver operating characteristic (ROC) analysis was calculated. AUC values of 0.5 indicate no predictive ability, values of 0.7–0.8 are considered to be acceptable, and values >0.8 are considered to be excellent. The greatest AUC indicates the most predictive model.

**Table 2:** Regression modeling of potential predictors of infection. Bold font indicates significance.

	Univariate analysis			Multivariable			Only clinical			Only lab values			Only lab values		
				analysis									+ Swab		
	p value	OR	95 % CI	p value	OR	95 % CI	p value	OR	95 % CI	p value	OR	95 % CI	p value	OR	95 % CI
Systemic	too few	too few	too few	too few	too few	too few									
Local	0.835624	0.89	0.3–2.9				0.299	0.298	0.02–2.817						
LocalRedCell	**0.06187**	3.54	1.05–16.3	0.7199	1.43	0.21–11.8	0.727	1.502	0.155–17.9						
StumpPain	**0.006853**	4.3	1.6–13.1	**0.04**	**7.2**	**1.29**–**65.78**	0.08	5.437	0.934–50.62						
Drainage	0.1308	2.3	0.82–6.94				0.598	0.585	0.069–4.28						
Discharge	0.114	3.5	0.88–23.9				0.25	5.134	0.44–166.31						
PainLoading	0.266875	0.58	0.22–1.53				0.11	5.55	0.77–66.9						
GrossMotion	**0.003274**	0.14	0.03–0.50	0.4	0.32	0.02–4.64	0.19	0.2	0.014–2.17						
CRP raw value	0.49106	1	0.99–1.03							0.07	1.1	1.02–1.26	0.06	1.14	1.02–1.36
ESR raw value	**0.052128**	0.98	0.95-0.99							0.014	0.94	0.88–0.98	0.01	0.93	0.87–0.97
WBC raw value	**0.01205**	1.4	1.1-1.99							0.14	1.39	0.92–2.32	0.09	1.53	0.96–2.77
CRP Binary	Does not fit	DNF	DNF												
ESR Binary	**0.045**	0.24	0.05–0.93	**0.04**	**0.13**	**0.01**–**0.75**									
WBC	0.15947	4.6	0.8–87												
>12000 µ L^-1^															
Swab	**0.056**	2.5	0.98–6.67	0.14	3.9	0.64–28.57							0.06	7.3	1.07–84.37

The data were organized using Google Sheets (Google LLC, Mountain View, California, USA). The analysis was performed using RStudio: Integrated Development for R (RStudio, PBC, Boston, MA URL http://www.rstudio.com/, last access: 31 January 2024) using the pROC package. The statistical significance was always set as p≤.05 .

## 3 Results

A total of 70 patients had 83 subsequent surgical episodes performed to manage suspected infection. The rate of positive intraoperative cultures was 57/83=69% ( 26/83=31% had no positive cultures). Table 1 presents the basic demographics of the two cohorts.

Table 2 presents the regression modeling of the primary outcome: potential predictors of positive cultures at subsequent surgery. As stated in the “Statistical analysis” section above, univariate regression modeling was performed as the first step in identifying associated independent variables. Several variables achieved statistical significance in univariate consideration. Associated with infection were stump pain ( p=.007 ), WBC >12000 per microliter ( p=.012 ), and positive swab from the peri-portal area ( p=.056 ). Associated with an aseptic situation were gross implant motion ( p=.003 ) and ESR >30 ( p=.045 ). The variable of cellulitis did not achieve statistically significant association with infection ( p=.062 ), but the decision was made to include it in further regression modeling because its inclusion yielded the best overall model. Pain was common in both cohorts but at a nearly significantly different proportion: 7/14=50% of non-infected patients had mechanically associated pain versus 53/69=77% of patients who had infection ( p=.054 ).

Upon performing the multivariable regression modeling, the following results were notable. Several variables identified on univariate analysis were no longer statistically significant: erythema or cellulitis, gross implant motion, and positive swab results. Residual limb pain remained significantly associated with infection ( p=.040 , OR =7.2 , 95 % CI =1.25 –65.78). An ESR >30 remained significantly associated against infection ( p=.040 , OR =.13 , 95 % CI =.01 –.75). When only clinical examination variables were considered, none were statistically significant. When only laboratory data or laboratory data with swab results were considered, increasing CRP approached significance of association with true infection, increasing ESR remained significant for association with an aseptic condition, and WBC remained not statistically significant; an isolated positive swab approached significant association with infection.

**Figure 2:**
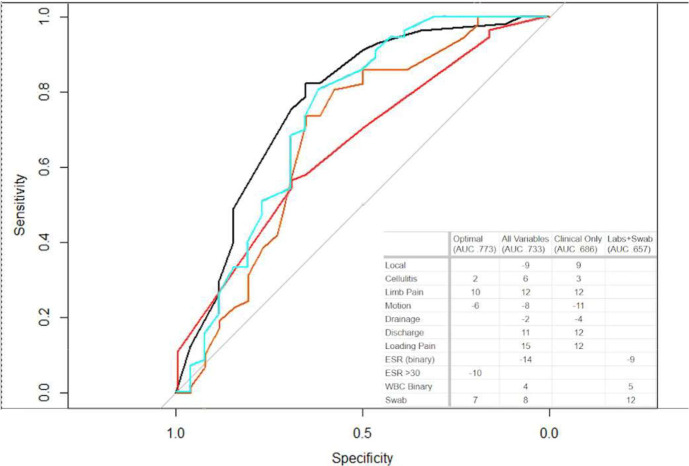
Area under receiver operator curve models. Four different models were created, as described in the main text. The black line indicates the optimal model. The turquoise line indicates all variables evaluated. The orange line indicates only clinical data. The red line indicates labs and swabs. The optimal model (black curve) achieved a positive predictive value of 85 %. Other models had less predictive reliability but are shown for relative utility comparison.

The next analysis performed to investigate the primary aim of variables associated with infection was AUC modeling. Figure 2 shows some featured models produced. The optimal AUC was achieved using the variables of cellulitis, residual limb pain, gross implant motion, ESR >30 , and a positive swab. In this model, 77 % of the surgical episodes could be correctly differentiated. Using this model's data, a scoring rubric was created, with each of the variables contributing relative weight to the total score (yielding calculated integer score coefficients). A score of ≥12 was identified to have a positive predictive value of 85 %. The variables most strongly associated with infection were stump pain (10) and a positive swab (7). Again, it is notable that elevated ESR >30 was associated with an aseptic situation rather than infection.

To understand the reliability of preoperatively swabbed cultures with intraoperatively swabbed cultures, Tables S1 and S2 in the Supplement were compiled based on the 57 surgeries which yielded positive intraoperative swab cultures. Table S1 identifies that preoperative swabs resulted positively for 42/57=74% of patients who eventually had a positive intraoperative culture. Further, 33/57=58% of patients had at least one intraoperative bacteria strain that was different from the preoperative swab strains. Restated, the swabs yielded false negatives for 26 % of patients with a deep infection. Antibiotics directed against bacteria cultured from peri-portal swabs would have been incorrect for 58 %.

Table S2 depicts the success of correctly identifying deep bacteria based on preoperative swab data. From the 57 operations with positive deep cultures, 88 bacteria strains grew. The most common bacteria category was various gram-negative species ( 22/88=25% ), followed by *Streptococcus* spp. ( 17/88=19% ), *Staphylococcus aureus* ( 16/88=18% ), various gram-positive species ( 14/88=16% ), *Pseudomonas* spp. ( 10/88=11% ), and *Escherichia coli* ( 9/88=10% ). A total of 40/88=45% of bacterial strains were not cultured from preoperative swabs. The most commonly missed type were various gram-negative species ( 11/40=28% ), followed by *Streptococcus* spp. ( 9/40=23% ), *Staphylococcus aureus* ( 6/40=15% ), various gram-positive species ( 5/40=13% ), *Pseudomonas* spp. ( 5/40=13% ), and *Escherichia coli* ( 4/40=10% ).

## 4 Discussion

The intent of this study was to create a diagnostic algorithm of peri-osseointegration infection. The best model estimates 85 % positive predictive value with a preoperative score of 12 or above. The most important findings of this study are that peri-implant limb pain is highly correlated with infection, ESR >30 is highly correlated against infection, a preoperative positive swab is moderately correlated with infection, gross implant motion is moderately correlated against infection, and erythema or cellulitis of the transcutaneous region is mildly correlated with infection. Many often-considered clinical variables (such as drainage or fevers) and laboratory values (such as CRP and WBC) are apparently inconsistently associated with deep infection for TOFA. It appears to be inappropriate to rely on elevated CRP to rule in infection or normal CRP to rule out infection.

TOFA has proven to be a revolutionary rehabilitation option for patients with limb loss. Being skeletally connected, the socket and thereby pathologic soft tissue compression are eliminated. Energy and momentum transfer from residual limb to prosthesis improves (Gaffney et al., 2022). Accordingly, reviews of the topic studies emphasize the overall substantial and often significant improvement in the mobility and quality of life of TOFA vs. SP (Hebert et al., 2017; Kunutsor et al., 2018). The most commonly reported complication remains infection (Reif et al., 2021; Atallah et al., 2018; Tillander et al., 2017; Gerzina et al., 2020), but no evidence-based algorithmic guidance exists to help diagnose infection prior to surgical intervention. As with any post-operative situation, infection can evolve even long after recovery normalizes (Brahme et al., 2023) so improved objective workup guidelines are important.

TOFA infection investigations generally retrospectively reported on care provided to patients with suspected infection (antibiotics or debridement with or without implant removal), such as the study of Tillander et al. (2010, 2017). In a 2018 review of TOFA complications, Atallah et al. (2018) identified that Al Muderis et al. (2016) were the first to publish a classification system correlating clinical, laboratory, and/or radiographic findings with the extent of intervention. A similar classification was subsequently published by Hoellwarth et al. (2022d). These studies may help understand the extent of infection management options or categorize research reporting, but they are not designed to guide diagnosis and management of patients presenting with concern of infection.

Other types of TOFA infection studies have surveyed the bacterial biome where the implant exits the skin. The portal is quickly colonized so positive cultures do not indicate infection (Tillander et al., 2010; Beck et al., 2019; Örgel et al., 2022). Tillander's 2010 study (Tillander et al., 2010) of 39 patients with 45 implants found that the most common colonizers were *Staphylococcus aureus*, coagulase-negative staphylococci, and streptococci groups. Evaluating 10 patients, Beck et al. (2019) also reported predominantly *Staphylococcus aureus*. The bacteria also changed over time; early post-operatively, there were more obligate anaerobes, changing over 1 year to mostly Streptococcus, Corynebacterium, and/or Staphylococcus. Evaluating 66 patients, Örgel et al. (2022) reported a similar high proportion of *Staphylococcus aureus*, *Staphylococcus* spp., and *Streptococcus* spp.. The current study exclusively evaluated patients suspected of infection rather than conducting routine surveillance. The most common peri-portal bacteria type was various gram-negative species ( 22/88=25% ), followed by *Streptococcus* spp. ( 17/88=19% ), *Staphylococcus aureus* ( 16/88=18% ), various gram-positive species ( 14/88=16% ), *Pseudomonas* spp. ( 10/88=11% ), and *Escherichia coli* ( 9/88=10% ). Rephrased, gram-negative species represented 41/88 =47% of skin portals suspected of infection, and gram-positive species represented 47/88=53% . This is a different biome than the routinely sampled patients, but it is unknown whether this shift is suggestive of infection. It is important to emphasize that it currently seems inappropriate to determine antibiotic coverage based on a preoperative swab. The antibiotics directed at these peri-portal bacteria would have been incorrect for 58 % of the cohort in this study. Basing empiric coverage for either gram-positive or gram-negative organisms is essentially no better than a random choice: 47 % versus 53 %. Further routine monitoring of the bacterial biome in the portal may eventually yield greater insight.

Joint replacement is a much more mature and high-volume field than TOFA, with more sophisticated infection research. Evidence-based PJI algorithms facilitate consistently improved guidance for diagnosis and management (Mühlhofer et al., 2017; Shohat et al., 2019) by confirming or refuting the diagnosis of PJI confidently and rapidly, expediting care, and avoiding unnecessary intervention. In the current study, 26/83=31% of surgeries performed to address suspected infection did not yield positive intraoperative cultures; a reliable algorithm may have avoided possibly unnecessary surgery. Current PJI literature emphasizes serological markers, typically minimally describing the history and physical aspects (Koh et al., 2015; Inman et al., 1984). But such objective information has not yet been elucidated for TOFA. TOFA patients with isolated peri-portal pain and temporally associated local erythema may have inflammation, irritation, or local cellulitis, all of which might be improved with a short period of reduced activity and/or oral or parenteral antibiotics. Pain deeper inside and occurring with weight bearing and/or new-onset drainage from the portal with changes in appearance, amount, or odor may suggest deep infection. A grossly loose implant without increasing drainage or odor suggests failure to integrate: removal and revision are still likely warranted and would probably be best with a prophylactic antibiotic depot, but in such cases, extensive debridement and soft tissue work might be avoidable.

PJI principles have informed other fields of orthopaedic infection diagnostics but must be applied with caution to TOFA. TOFA implants are transcutaneous into the bone, seemingly permanently fulfilling the criterion of a sinus tract; the vast majority of TOFA patients are not infected. The second criterion of two identical peri-implant aspiration cultures seems unable to be implemented as there is no apparent ability to aspirate between the implant and the bone. As aforementioned, external peri-portal swabbing will frequently culture external flora regardless of infection status, though longitudinal studies may reveal floral shifts from healthy to infected states. Elevated ESR and CRP were not associated with infection in the current study. The authors do not have a satisfying explanation for this lack of inflammatory marker elevation for TOFA infection. A straightforward statement is that it seems that inflammation is not consistently and/or strongly elicited in this situation, at least as assessed by ESR and CRP. Another possibility could be that the open portal allows some drainage of infection, whereas PJI generally keeps the infection sealed until a possible eventual sinus eruption. It is emphasized that these proposals have not been investigated. Further direct research is necessary to elucidate potentially reliable serologic markers.

This study's most important limitation is sample size. Representing 83 surgeries on 70 patients performed for suspected infection, with 57 yielding positive cultures, the data are far fewer than the many thousands utilized for PJI studies. But TOFA does not have such volume. The relative strength in this regard is that this study's cohort comes from the world's largest TOFA registry. A second limitation is the retrospective data analysis; there may be reporting bias regarding history elicited, physical evaluations, and changes in clinicians' judgments over time. To minimize diagnostic bias, this study's criteria for infection was positive intraoperative cultures. Finally, it is emphasized that this study is not designed or promoted as a definitive study of peri-TOFA infection. Rather, as the first study of its kind, the intent is to serve as a foundation. It already seems apparent that a broader range of potential lab markers is necessary.

## 5 Conclusions

This seminal investigation to develop a preoperative diagnostic algorithm for peri-TOFA infection revealed that history and physical examination remain the most essential considerations. Peri-implant pain, particularly with an apparent local cellulitis, is highly suggestive of infection, whereas inflammatory markers appear to be non-contributory. Further evaluation of a wider spectrum of clinical, laboratory, and imaging data, consistently and routinely collected with prospective data techniques in larger cohorts of patients, are necessary and encouraged.

## Supplement

10.5194/jbji-9-49-2024-supplement The supplement related to this article is available online at: https://doi.org/10.5194/jbji-9-49-2024-supplement.

## Data Availability

The data that support the findings of this study are available on request from the corresponding author. The data are not publicly available due to privacy or ethical restrictions.
