# Supplementary material for: Development of an evidence-based diagnostic algorithm for infection in patients with transcutaneous osseointegration following amputation
Source: J Bone Jt Infect. 2024 Feb 6;9(1):49–57. doi: 10.5194/jbji-9-49-2024 (PMC11002918; doi:10.5194/jbji-9-49-2024)
Supplement: The supplement related to this article is available online at: https://doi.org/10.5194/jbji-9-49-2024-supplement. [file jbji-9-49-supplement.pdf]

Supplement of J. Bone Joint Infect., 9, 49–57, 2024
https://doi.org/10.5194/jbji-9-49-2024-supplement
© Author(s) 2024. CC BY 4.0 License.

Supplement of

Development of an evidence-based diagnostic algorithm for infection in patients with transcutaneous osseointegration following amputation

Shafaf Hasin Alam et al.

Correspondence to: Jason S. Hoellwarth (drjsoon@gmail.com)

The copyright of individual parts of the supplement might differ from the article licence.

Table S1 - List of entire bacteria culture results, correlated preoperative with postoperative.

Episode	Any Intraoperative Culture Different from Preoperative Swab	Negative Preoperative Swab, Positive Surgery	Pre1	Intra1	Pre2	Intra2	Pre3	Intra3	Intra4
1	1	1	-	Staphylococcus aureus (MRSA)					
2	1		Group G Streptococcus	Bacillus circulans					
3			Staphylococcus epidermidis	Staphylococcus epidermidis					
4			Staphylococcus aureus	Staphylococcus aureus					
5	1	1	-	Morganella morganii					
6			Staphylococcus aureus	Staphylococcus aureus					
7	1	1	-	Staphylococcus aureus		Streptococcus intermedius (SAG)		Staphylococcus epidermidis	
8	1	1	-	Enterococcus faecalis					
9	1		Streptococcus intermedius (SAG)	Streptococcus intermedius (SAG)		Escherichia coli			
10	1		Streptococcus intermedius (SAG)	Streptococcus intermedius (SAG)		Escherichia coli			
11			-	*					
12			Group B Streptococcus	Group B Streptococcus					
13	1	1	-	Group B Streptococcus		Bacteroides fragilis			
14	1	1	-	Staphylococcus aureus					
15			Enterobacter clocae	Enterobacter clocae					
16			Serratia marcescens	Serratia marcescens	Morganella morganii	Morganella morganii	Enterococcus faecalis	Enterococcus faecalis	

17			Staphylococcus aureus	Staphylococcus aureus					
18			-	*					
19	1		Staphylococcus aureus	Staphylococcus aureus		Streptococcus agalactiae			
20	1	1	-	Staphylococcus aureus		Serratia marcescens		Pseudomonas aeruginosa	
21			-	*					
22			Group B Streptococcus	Group B Streptococcus					
23	1	1	-	Escherichia coli		Streptococcus anginosus (SAG)		Pasteurella canis	
24	1	1	-	Staphylococcus aureus					
25	1		Gram positive cocci	Staphylococcus epidermidis					
26	1		Staphylococcus epidermidis	Staphylococcus epidermidis		Morganella morganii			
27			Psuedomonas aeruginosa	Pseudomonas aeruginosa					
28	1		Group G Streptococcus	Pseudomonas aeruginosa	Proteus mirabilis	Proteus vulgaris		Bacteroides fragilis	
29	1	1	-	Staphylococcus epidermidis					
30			-	*					
31			Staphylococcus aureus	Serratia marcescens					
32	1		Staphylococcus aureus	Serratia marcescens		Pseudomonas aeruginosa		Streptococcus agalactiae (Group B)	cus anginc
33	1		Staphylococcus aureus	Staphylococcus aureus		Pseudomonas aeruginosa			
34	1		Staphylococcus aureus	Staphylococcus aureus		Enterococcus faecalis			
35			Staphylococcus aureus (MRSA)	Staphylococcus aureus (MRSA)					
36	1		Escherichia coli	Escherichia coli	Streptococcus milleri (SAG)	Streptococcus milleri (SAG)		Pseudomonas aeruginosa	

37	1		Streptococcus milleri (SAG)	Streptococcus milleri (SAG)		Streptococcus constellatus (SAG)			
38	1	1	-	Escherichia coli		Klebsiella pneumoniae		Streptococcus constellatus (SAG)	
39	1	1	-	Eikenella corrodens		Bacteroides species			
40	1		Streptococcus species (SAG)	Propionibacterium acnes					
41			Staphylococcus aureus (MRSA)	Staphylococcus aureus (MRSA)		Enterobacter clocae			
42			Enterobacter clocae	Enterobacter clocae					
43	1		Staphylococcus aureus	Klebsiella oxytoca					
44	1		Pseudomonas	Pseudomonas aeruginosa	Streptococcus pyogenes	Streptococcus anginosus (SAG)			
45	1		Escherichia coli	Escherichia coli	Enterococcus faecalis	Enterococcus faecalis		Pseudomonas	
46	1	1	-	Staphylococcus aureus					
47	1		Staphylococcus aureus	Staphylococcus aureus	Escherichia coli	Escherichia coli		Klebsiella pneumoniae	
48			Escherichia coli	Escherichia coli					
49			Streptococcus milleri (SAG)	Streptococcus milleri (SAG)					
50	1	1	-	Actinotignum schaalii					
51			Staphylococcus aureus	Staphylococcus aureus					
52			Staphylococcus aureus	Enterococcus faecalis	Pseudomonas aeruginosa	Pseudomonas aeruginosa	Aeromonas		
53			Pseudomonas aeruginosa	Pseudomonas aeruginosa	Enterococcus faecalis				
54			Proteus mirabilis	Proteus mirabilis	Escherichia coli				
55	1		Escherichia coli	Enterococcus faecium	Acinetobacter	Stenotrophomonas maltophilia	Klebsiella pneumonia		
56	1		Pseudomonas aeruginosa	Proteus mirabilis		Streptococcus agalactiae (Group B)			

57			Escherichia coli	Escherichia coli					

This table identifies all the bacteria identified by culture swabs.
*These four patients did not have the original operative culture result data available to determine the specific bacteria; however, they had postoperative follow-up notes stating the operative culture was positive. Therefore, they could be certain to have positive intraoperative bacterial cultures, even if the specific species could not be determined retrospectively.

Table S2 - success of preoperative swab correctly identifying intraoperative cultures

Preoperative Swab	Intraoperative Culture	0=Negative Intraoperative<br>1=Correct<br>2=Gram Correct<br>3=Gram Wrong<br>4=Preoperative Negative
Aeromonas		0
Klebsiella pneumonia		0
Enterobacter clocae	Enterobacter clocae	1
Enterobacter clocae	Enterobacter clocae	1
Morganella morganii	Morganella morganii	1
Proteus mirabilis	Proteus mirabilis	1
Serratia marcescens	Serratia marcescens	1
Acinetobacter	Stenotrophomonas maltophilia	2
Proteus mirabilis	Proteus vulgaris	2
Enterococcus faecalis		0
Enterococcus faecalis	Enterococcus faecalis	1
Enterococcus faecalis	Enterococcus faecalis	1
Staphylococcus epidermidis	Staphylococcus epidermidis	1
Staphylococcus epidermidis	Staphylococcus epidermidis	1
Gram positive cocci	Staphylococcus epidermidis	2
Escherichia coli		0
Escherichia coli	Escherichia coli	1
Escherichia coli	Escherichia coli	1
Escherichia coli	Escherichia coli	1
Escherichia coli	Escherichia coli	1
Escherichia coli	Escherichia coli	1
Escherichia coli	Enterococcus faecium	3
Group B Streptococcus	Group B Streptococcus	1
Group B Streptococcus	Group B Streptococcus	1
Streptococcus intermedius (SAG)	Streptococcus ontermedius (SAG)	1
Streptococcus intermedius (SAG)	Streptococcus ontermedius (SAG)	1
Streptococcus milleri (SAG)	Streptococcus milleri (SAG)	1
Streptococcus milleri (SAG)	Streptococcus milleri (SAG)	1
Streptococcus milleri (SAG)	Streptococcus milleri (SAG)	1
Group G Streptococcus	Bacillus circulans	2
Streptococcus species (SAG)	Propionibacterium acnes	2
Streptococcus pyogenes	Streptococcus anginosus (SAG)	2
Group G Streptococcus	Pseudomonas aeruginosa	3

Pseudomonas	Pseudomonas aeruginosa	1
Pseudomonas aeruginosa	Pseudomonas aeruginosa	1
Pseudomonas aeruginosa	Pseudomonas aeruginosa	1
Psuedomonas aeruginosa	Pseudomonas aeruginosa	1
Pseudomonas aeruginosa	Proteus mirabilis	2
Staphylococcus aureus	Staphylococcus aureus	1
Staphylococcus aureus	Staphylococcus aureus	1
Staphylococcus aureus	Staphylococcus aureus	1
Staphylococcus aureus	Staphylococcus aureus	1
Staphylococcus aureus	Staphylococcus aureus	1
Staphylococcus aureus	Staphylococcus aureus	1
Staphylococcus aureus	Staphylococcus aureus	1
Staphylococcus aureus	Staphylococcus aureus	1
Staphylococcus aureus (MRSA)	Staphylococcus aureus (MRSA)	1
Staphylococcus aureus	Enterococcus faecalis	2
Staphylococcus aureus	Klebsiella oxytoca	3
Staphylococcus aureus	Serratia marcescens	3
Staphylococcus aureus	Serratia marcescens	3
Staphylococcus aureus (MRSA)	Staphylococcus aureus (MRSA)	1
	Bacteroides	4
	Bacteroides fragilis	4
	Bacteroides fragilis	4
	Eikenella corrodens	4
	Enterobacter clocae	4
	Klebsiella pneumoniae	4
	Klebsiella pneumoniae	4
	Morganella morganii	4
	Morganella morganii	4
	Pasteurella canis	4
	Serratia marcescens	4
	Actinotignum schaalii	4
	Enterococcus faecalis	4
	Enterococcus faecalis	4
	Staphylococcus epidermidis	4
	Staphylococcus epidermidis	4
	Escherichia coli	4
	Escherichia coli	4
	Escherichia coli	4
	Escherichia coli	4

	Group B Streptococcus	4
	Streptococcus agalactiae	4
	Streptococcus agalactiae	4
	Streptococcus agalactiae	4
	Streptococcus anginosus (SAG)	4
	Streptococcus anginosus (SAG)	4
	Streptococcus constellatus (SAG)	4
	Streptococcus constellatus (SAG)	4
	Streptococcus intermedius (SAG)	4
	Pseudomonas	4
	Pseudomonas aeruginosa	4
	Pseudomonas aeruginosa	4
	Pseudomonas aeruginosa	4
	Pseudomonas aeruginosa	4
	Staphylococcus aureus	4
	Staphylococcus aureus	4
	Staphylococcus aureus	4
	Staphylococcus aureus	4
	Staphylococcus aureus	4
	Staphylococcus aureus (MRSA)	4

This table compares the bacteria cultured from the preoperative swab versus the bacteria that was grown from deep operative cultures. Color highlighting of the first two columns is provided to aid in understanding the bacterial types: red=other gram negative, orange=other gram positive, yellow=Escherichia coli, green=streptococcus species, blue=pseudomonas species, magenta=staphylococcus aureus. The third column identifies the correctness of preoperative swab with intraoperative culture; the text color coding is intended to estimate the potential degree of concern of any mismatch; black (0)=swab culture without operative culture isolated, green (1)=matched swab and operative culture, orange (2)=incorrect cultured species but correct gram status, magenta (3)=incorrect culture and incorrect gram status, red (4)=undetected on preoperative swab
